# Supplementary material for: Which Seed Properties Determine the Preferences of Carabid Beetle Seed Predators?
Source: Insects. 2020 Nov 4;11(11):757. doi: 10.3390/insects11110757 (PMC7692740; doi:10.3390/insects11110757)
Supplement: Supplementary file 1 [file insects-11-00757-s001.pdf]

Supplementary materials

Foffová H., Ćavar Zeljković S., Honěk A. , Martinkova Z., Tarkowski P., Saska P.: Which seed properties determine the preferences of carabid beetle seed predators?

Appendix S1

Content tables of results:

Table 1 (*Preferences of carabids*)……………………………………………………...…………1 - 3
Table 2 (*Morphological properties of the seeds*)…………………………………………………….……4
Table 3 (*Surface fatty acids from seeds*)……………………………………………………………5 - 6
Table 4 (*Total fatty acids from seeds*)…………………………………………………………….. 7 - 8
Table 5 (*Volatiles from imbibed seeds*)…………………………………….…………………………...9
Table 6 (*Volatiles from dry seeds*)…………………………………………………….………………10
Table 7 (*Other surface compounds*)………………………………………………………………… 11

Appendix S1: Table 1. The standardized consumption of 37 carabid species on 28 seed species. The preferences were determined based on a cafeteria experiment described in Honěk et al. [6] and Saska et al. [7].

**Species**	*Acupalpus meridianus* (Linnaeus)	*Amara aenea* (DeGeer)	*Amara anthobia* (A. Villa et G.B. Villa)	*Amara apricaria* (Paykull)	*Amara aulica* (Panzer)	*Amara bifrons* (Gyllenhal)	*Amara consularis* (Duftschmid)	*Amara convexior* (Stephens)	*Amara convexiuscula* (Marsham)	*Amara eurynota* (Panzer)	*Amara familiaris* (Duftschmid)	*Amara ingenua* (Duftschmid)
Amaranthus retroflexus L.	0.016	0.002	0.002	0	0.065	0.002	0.085	0.012	0.089	0.075	0	0.011
Arctium lappa L.	0	0.003	0	0	0.17	0	0.013	0.003	0.026	0.005	0	0.011
Arenaria serpyllifolia agg.	0.295	0.508	0.05	0.006	0.053	0.012	0.039	0.305	0.04	0.052	0.4	0.055
Bidens tripartita L.	0	0	0	0	0.029	0	0	0.021	0.092	0.018	0	0.075
Campanula trachelium L.	0.262	0.444	0.031	0.175	0.031	0.044	0.876	0.994	0.322	0.191	0.103	0.018
Capsella bursa-pastoris (L.) Med.	0.557	1	0.565	0.038	0.033	0.194	0.163	0.103	0.071	0.54	0.526	0.114
Chenopodium album L.	0	0	0	0	0.01	0	0.007	0.003	0.106	0.004	0.002	0.088
Cichorium intybus L.	0	0	0	0.031	0.436	0	0.039	0.015	0.386	0.107	0.015	0.471
Cirsium arvense (L.) Scop.	0.016	0.022	0.015	0.319	1	0.021	0.712	0.032	0.393	0.263	0.01	0.713
Consolida regalis S.F.Gray	0	0	0	0	0.014	0.002	0	0	0.087	0.005	0.01	0.015
Crepis biennis L.	0.016	0.071	0.015	0.144	0.031	0.028	0.137	0.1	0.301	0.066	0.006	0.156
Descurainia sophia (L.) Prantl	0.738	0.813	0.379	0	0.065	0.052	0.039	0.314	0.191	0.465	0.333	0.055
Fumaria officinalis L.	0	0	0	0	0	0	0.007	0	0.002	0.01	0	0
Galinsoga parviflora Cav.	0.098	0.131	0.042	0.019	0.043	0.096	0.013	0.402	0.518	0.2	0.064	0.215
Galium aparine L.	0	0	0	0.006	0.015	0	0.007	0.006	0.024	0	0	0.002
Lapsana communis L.	0	0	0	0	0.132	0	0.033	0.006	0.127	0.041	0.01	0.046
Leonurus cardiaca L.	0	0	0.003	0.019	0.065	0.005	0.02	0.006	0.064	0.074	0.01	0.011
Lepidium ruderale L.	0.459	0.744	0.421	0.063	0.01	0.026	0.013	0.375	0.111	0.615	0.329	0.175
Melilotus albus Med.	0.033	0	0	0.019	0.003	0	0.02	0.006	0.04	0.016	0	0.009
Potentilla argentea L.	0.066	0.036	0.009	0.019	0.024	0.016	0.059	0.158	0.266	0.086	0.043	0.033
Silene latifolia alba (Mill.) Greut. et Burdet	0.033	0.204	0.178	0.144	0.033	0.023	0.275	0.179	0.412	0.295	0.516	0.441
Sisymbrium loeselii L.	1	0.055	0.063	0	0.07	0.11	0.02	0.023	0.122	0.243	0.062	0.029
Stellaria media (L.) Vill.	0.033	0.784	1	0.356	0.086	0.061	0.51	0.094	0.101	0.517	1	0.05
Taraxacum officinale agg.	0.098	0.907	0.325	1	0.16	1	1	0.507	1	1	0.151	1
Thlapsi arvense L.	0	0.026	0	0.063	0.01	0.009	0.196	0.073	0.087	0.373	0.004	0.044
Tripleurospermum inodorum (L.) Schu.-Bip.	0.016	0.295	0.002	0	0.149	0.044	0.111	1	0.946	0.532	0.041	0.743
Urtica dioica L.	0.016	0.043	0.06	0.006	0.043	0.049	0.078	0.883	0.32	0.235	0.118	0.474
Viola arvensis Murray	0.016	0.014	0.003	0	0.046	0.012	0.124	0.062	0.278	0.16	0.004	0.259

Appendix S1: Table 1. (Continued)

Species	*Amara litorea* (C.G.Thomson)	*Amara montivaga* (Sturm)	*Amara ovata* (Fabricius)	*Amara sabulosa* (Audient-Serville)	*Amara similata* (Gyllenhal)	*Amara spreta* (Dejean)	*Anisodactylus signatus* (Panzer)	*Calathus ambiguus* (Paykull)	*Calathus fuscipes* (Goeze)	*Harpalus affinis* (Schrank)	*Harpalus atratus* (Latreille)	*Harpalus distinguendus* (Duftschmid)
Amaranthus retroflexus L.	0.001	0	0.014	0	0.01	0	0.285	0	0.051	0.206	0.582	0.127
Arctium lappa L.	0.006	0	0.006	0	0	0	0.005	0	0	0.022	0.021	0.01
Arenaria serpyllifolia agg.	0.061	0.004	0.086	0.022	0.41	0.122	0.056	0.028	0.949	0.037	0.011	0.038
Bidens tripartita L.	0.007	0.003	0	0	0.003	0.006	0.173	0	0	0.087	0	0.084
Campanula trachelium L.	0.45	0.001	0.044	0.086	0.608	0.04	0.307	0	0.59	0.117	0.073	0.241
Capsella bursa-pastoris (L.) Med.	0.901	0.004	0.832	0.129	0.986	0.627	0.138	0.222	0.41	0.269	0.201	0.309
Chenopodium album L.	0.006	0	0	0	0.003	0.005	0.491	0.028	0	0.128	0.301	0.249
Cichorium intybus L.	0.027	0.017	0.015	0.011	0.003	0.018	0.853	0	0	0.586	0.466	0.446
Cirsium arvense (L.) Scop.	0.07	0.001	0.181	0	0.039	0.012	0.913	0	0.333	0.72	0.655	0.528
Consolida regalis S.F.Gray	0.003	0	0.014	0	0.004	0.005	0.116	0.111	0.051	0.093	0.005	0.026
Crepis biennis L.	0.024	1	0.035	0.113	0.067	0.075	0.354	0	0.026	0.165	0.009	0.203
Descurainia sophia (L.) Prantl	0.984	0.017	0.677	0.108	0.961	0.786	0.096	0	1	0.137	0.089	0.388
Fumaria officinalis L.	0	0	0.012	0	0	0	0.023	0	0	0.004	0.073	0.003
Galinsoga parviflora Cav.	0.109	0.001	0.039	0.495	0.09	0.679	0.757	0.028	0.179	0.269	0.098	0.698
Galium aparine L.	0	0	0.008	0	0	0	0.006	0	0.026	0.026	0.009	0.002
Lapsana communis L.	0.006	0.016	0.009	0.027	0.003	0	0.285	0	0	0.113	0.027	0.109
Leonurus cardiaca L.	0.016	0.001	0.027	0	0.003	0.032	0.406	0.028	0.282	0.232	0.658	0.219
Lepidium ruderale L.	0.648	0.007	0.472	0.161	1	0.299	0.113	0.361	0.179	0.093	0.176	0.558
Melilotus albus Med.	0.001	0	0.002	0	0.001	0	0.04	0.139	0.308	0.033	0.032	0.018
Potentilla argentea L.	0.018	0.001	0.027	0	0.038	0.012	0.238	0	0.282	0.085	0.126	0.114
Silene latifolia alba (Mill.) Greut. et Burdet	0.172	0.037	0.258	0.022	0.213	0.215	0.61	0	0.077	0.334	0.057	0.533
Sisymbrium loeselii L.	0.638	0.003	0.467	0.005	0.622	0.072	0.094	0	0.026	0.289	0.055	0.048
Stellaria media (L.) Vill.	0.256	0.003	0.428	0.032	0.856	0.279	0.133	0	0.769	0.169	0.418	0.14
Taraxacum officinale agg.	0.5	0.993	1	1	0.931	1	1	1	0.128	0.761	0.057	0.764
Thlapsi arvense L.	1	0.007	0.836	0	0.699	0.009	0.296	0	0.026	0.26	0.103	0.147
Tripleurospermum inodorum (L.) Schu.-Bip.	0.051	0.053	0.115	0.645	0.439	0.571	0.757	0	0.564	0.805	0.08	1
Urtica dioica L.	0.469	0.001	0.589	0.048	0.509	0.133	0.644	0	0.026	0.49	0.105	0.526
Viola arvensis Murray	0.044	0.005	0.071	0.016	0.043	0.023	0.655	0	0	1	1	0.761

Appendix S1: Table 1. (Continued)

Species	*Harpalus honestus* (Duftschmid)	*Harpalus luteicornis* (Duftschmid)	*Harpalus rubripes* (Duftschmid)	*Harpalus signaticornis* (Duftschmid)	*Harpalus subcylindricus* (Dejean)	*Ophonus azureus* (Fabricius)	*Parophonus maculicornis* (Duftschmid)	*Pseudoophonus griseus* (Panzer)	*Pseudoophonus rufipes* (DeGeer)	*Pterostichus melanarius* (Illiger)	*Stenolophus teutonus* (Schrank)	*Trechus quadristriatus* (Schrank)	*Zabrus tenebrioides* (Goeze)
Amaranthus retroflexus L.	0.377	0.035	0.051	0.052	0.119	0	0.011	0.798	0.42	0.08	0	0	0.003
Arctium lappa L.	0.014	0	0.004	0	0	0	0	0.003	0.058	0.025	0	0	0.006
Arenaria serpyllifolia agg.	0.114	0.058	0.011	0.194	0.129	0	0.193	0.059	0.01	0.012	0.66	0.079	0.058
Bidens tripartita L.	0	0.006	0.056	0.002	0	0	0	0.006	0.066	0.056	0.005	0	0.108
Campanula trachelium L.	0.18	0.099	0.103	0.12	0.465	0.061	0.239	0.354	0.082	0.123	0.447	0.079	0.006
Capsella bursa-pastoris (L.) Med.	0.474	0.362	0.027	0.442	0.505	0.182	0.091	0.156	0.129	0.037	1	1	0.02
Chenopodium album L.	0.054	0.061	0.458	0.014	0	0.045	0	0.445	0.256	0.056	0	0	0.024
Cichorium intybus L.	0.106	0.076	1	0.056	0.035	0.015	0	0.522	0.908	0.049	0	0	0.406
Cirsium arvense (L.) Scop.	0.64	0.434	0.658	0.192	0.04	0.258	0	0.739	1	1	0	0	0.187
Consolida regalis S.F.Gray	0.003	0	0.004	0.025	0	0	0	0.023	0.05	0.049	0	0	0.055
Crepis biennis L.	0.023	0.283	0.1	0.047	0.193	0	0	0.029	0.043	0.216	0	0	0.265
Descurainia sophia (L.) Prantl	0.217	0.152	0.051	0.33	1	0.212	0.67	0.304	0.045	0.006	0.699	0.19	0.011
Fumaria officinalis L.	0.023	0	0.208	0	0	0	0	0.046	0.042	0.031	0	0	0.004
Galinsoga parviflora Cav.	0.209	0.329	0.241	0.53	0.832	0.061	1	0.933	0.169	0.074	0.316	0	0.034
Galium aparine L.	0.003	0	0.002	0.002	0	1	0	0.003	0.006	0.006	0	0	0.001
Lapsana communis L.	0.023	0.073	0.011	0.018	0.015	0	0	0.042	0.056	0.093	0	0	0.035
Leonurus cardiaca L.	0.463	0.12	0.076	0.072	0.074	0	0	0.214	0.127	0.012	0.005	0	0.041
Lepidium ruderale L.	0.28	0.146	0.069	0.174	0.282	0.015	0.511	0.488	0.069	0.019	0.49	0.444	0.02
Melilotus albus Med.	0.037	0	0.422	0	0	0.045	0	0.118	0.089	0.006	0	0	0.003
Potentilla argentea L.	0.054	0.108	0.123	0.223	0.089	0.015	0.17	0.71	0.027	0.006	0.34	0	0.01
Silene latifolia alba (Mill.) Greut. et Burdet	0.074	0.303	0.107	0.072	0.02	0.121	0.125	0.257	0.227	0.025	0.117	0	0.579
Sisymbrium loeselii L.	0.071	0.076	0	0.104	0.743	0.136	0.034	0.042	0.082	0.062	0.141	0.841	0
Stellaria media (L.) Vill.	0.503	0.163	0.132	0.12	0.114	0.015	0.193	0.08	0.072	0.074	0.505	0.032	0.03
Taraxacum officinale agg.	0.114	0.589	0.353	0.454	0.698	0.061	0.341	0.323	0.303	0.278	0.16	0	1
Thlapsi arvense L.	0.037	0.07	0.027	0.126	0.01	0.273	0.023	0.088	0.156	0.012	0	0	0.122
Tripleurospermum inodorum (L.) Schu.-Bip.	0.2	0.245	0.241	0.196	0.619	0.758	0	0.063	0.201	0	0.029	0	0.267
Urtica dioica L.	0.309	0.198	0.158	0.151	0.609	0	0.432	0.971	0.573	0.062	0.388	0.048	0.011
Viola arvensis Murray	1	1	0.967	1	0.743	0.015	0.159	1	0.995	0.315	0.019	0	0.003

Appendix S1: Table 2. The morphological properties of the seeds used in preference experiment. Botanical nomenclature is based on Kubát et al. [58]. Classification of plant strategy (A - annual, B - biennial, A-B - annual or biennial, P – perennial plants) is based on Bojnanský and Fargašová [63]. The mass of the seeds is based on measurements of 100 seeds. Indexes of seed shape are based on Cerda et al. [59] and Bekker et al. [38]. The variation between mechanical defence in coat strength and seed coat thickness.

**Species**	**Strategy**	**Mass [g]**	**A [mm]**	**B [mm]**	**C [mm]**	**Shape index**	**Flatness index**	**Excentricity index**	**Volume**	**Seed strength [N]**	**Seed coat thickness [mm]**
Amaranthus retroflexus L.	A	0.53	1.172±0.118	0.984±0.079	0.63±0.053	0.037±0.002	1.712±0.041	1.19±0.025	0.741±0.09	19.95±1.299	0.048±0.005
Arctium lappa L.	B	8.72	5.63±0.028	2.308±0.23	1.302±0.035	0.108±0.002	3.05±0.059	2.466±0.134	16.932±0.926	53.67±15.61	0.134±0.069
Arenaria serpyllifolia agg.	A-B	0.05	0.552±0.116	0.39±0.041	0.272±0.372	0.045±0.01	1.737±0.097	1.451±0.224	0.058±0.006	4.86±0.518	0.138±0.043
Bidens tripartita L.	A	2.69	9.076±1.264	2.036±0.131	0.414±0.043	0.171±0.002	13.496±0.73	4.47±0.313	7.698±0.842	13.43±1.268	0.076±0.01
Campanula trachelium L.	A-B	0.18	1.188±0.056	0.618±0.06	0.294±0.055	0.097±0.003	3.089±0.131	1.935±0.077	0.217±0.019	1.5±0.755	0.037±0.002
Capsella bursa-pastoris (L.) Med.	A-B	0.23	1.09±0.047	0.59±0.067	0.41±0.065	0.071±0.006	2.097±0.166	1.87±0.107	0.268±0.037	1.84±0.533	0.019±0.004
Cichorium intybus L.	P	1.09	2.532±0.124	1.21±0.057	0.572±0.134	0.104±0.004	3.352±0.253	2.094±0.042	1.765±0.196	7.83±3.693	0.069±0.016
Cirsium arvense (L.) Scop.	P	0.79	2.824±0.086	1.084±0.065	0.572±0.045	0.117±0.002	3.43±0.096	2.612±0.064	1.761±0.132	6.85±1.708	0.081±0.012
Consolida regalis S.F.Gray	A	1.38	1.93±0.155	1.164±0.114	1.04±0.216	0.042±0.006	1.499±0.076	1.674±0.101	2.354±0.229	6.85±1.388	0.027±0.004
Crepis biennis L.	B	0.67	4.17±0.495	0.66±0.036	0.572±0.028	0.161±0.003	4.237±0.265	6.319±0.322	1.576±0.123	5.35±1.363	0.044±0.01
Descurainia sophia (L.) Prantl	A	0.11	0.792±0.074	0.4±0.067	0.364±0.089	0.061±0.007	1.637±0.029	2.032±0.194	0.117±0.017	1.55±0.201	0.045±0.008
Fumaria officinalis L.	A	3.01	1.694±0.116	1.572±0.043	1.434±0.072	0.005±0.003	1.144±0.052	1.077±0.032	3.81±0.11	4.23±1.134	0.121±0.011
Galinsoga parviflora Cav.	A	0.17	1.658±0.289	0.524±0.144	0.35±0.418	0.123±0.009	3.203±0.232	3.334±0.404	0.331±0.092	1.86±0.463	0.029±0.003
Galium aparine L.	A	6.64	2.886±0.262	2.578±0.332	1.688±0.281	0.032±0.004	1.634±0.065	1.125±0.029	13.096±2.503	99.47±16.818	0.017±0.006
Chenopodium album L.	A	0.7	1.24±0.048	1.17±0.045	0.628±0.304	0.049±0.002	1.922±0.032	1.06±0.019	0.915±0.054	17.39±3.75	0.078±0.016
Lapsana communis L.	A	1.38	3.47±0.118	1.04±0.12	0.506±0.101	0.138±0.005	4.648±0.496	3.391±0.24	1.824±0.214	14.83±1.812	0.099±0.006
Leonurus cardiaca L.	P	0.64	2.264±0.196	1.398±0.058	0.816±0.137	0.069±0.004	2.262±0.098	1.617±0.047	2.614±0.272	3.35±1.112	0.065±0.008
Lepidium ruderale L.	A-B	0.21	1.55±0.123	1.2±0.12	0.412±0.026	0.095±0.003	3.34±0.114	1.297±0.045	0.775±0.08	7.34±3.007	0.062±0.004
Melilotus albus Med.	P	0.79	1.942±0.09	1.454±0.103	1.006±0.105	0.039±0.004	1.7±0.067	1.339±0.03	2.872±0.298	29.97±5.77	0.061±0.01
Potentilla argentea L.	P	0.08	1.178±0.121	0.948±0.061	0.756±0.152	0.023±0.002	1.412±0.039	1.249±0.084	0.85±0.083	1.53±0.269	0.079±0.016
Silene latifolia alba (Mill.) Greut. et Burdet	A-B	1.81	0.796±0.046	0.682±0.023	0.598±0.118	0.012±0.003	1.246±0.058	1.167±0.025	0.326±0.023	29.59±5.234	0.042±0.007
Sisymbrium loeselii L.	A-B	0.08	0.99±0.206	0.572±0.079	0.466±0.021	0.052±0.013	1.684±0.128	1.788±0.28	0.261±0.027	1.16±0.484	0.02±0.003
Stellaria media (L.) Vill.	A-B	0.42	1.05±0.035	1.02±0.037	0.626±0.063	0.034±0.002	1.655±0.034	1.03±0.012	0.672±0.031	17.52±3.049	0.035±0.008
Taraxacum officinale agg.	P	0.48	2.88±0.266	0.916±0.064	0.322±0.067	0.144±0.003	6.094±0.517	3.168±0.213	0.862±0.124	1.3±0.621	0.067±0.017
Thlapsi arvense L.	A-B	0.97	2.052±0.182	1.392±0.132	0.748±0.182	0.068±0.004	2.32±0.097	1.478±0.043	2.185±0.282	11.53±2.265	0.04±0.003
Tripleurospermum inodorum (L.) Schultz-Bip.	A	0.32	1.89±0.036	0.708±0.072	0.626±0.055	0.094±0.004	2.089±0.088	2.695±0.129	0.842±0.073	5.89±1.527	0.048±0.003
Urtica dioica L.	P	0.14	1.18±0.094	0.76±0.043	0.368±0.128	0.079±0.002	2.649±0.088	1.552±0.041	0.334±0.034	1.14±0.533	0.056±0.021
Viola arvensis Murray	A	0.46	1.572±0.099	0.92±0.08	0.826±0.062	0.045±0.005	1.515±0.065	1.717±0.067	1.203±0.109	2.49±0.601	0.049±0.006

Appendix S1: Table 3. The amount of total fatty acids. The fatty acids were isolated and derivatized into corresponding volatile methyl esters and then quantified via GC-MS method. The highest content of fatty acids was found in the seeds of *G. parviflora* (467.75±8.40 mg/g$_{DW}$), while the smallest was in the seeds of *G. aparine* (33.39 ±1.26 mg/g$_{DW}$). The major fatty acid in all seeds analyzed was unsaturated linoleic acid, which amount comprised ~50% of total fatty acids quantified.

Species	Octanoic	Decanoic	Undecanoic	Dodecanoic	Myristoleic	Tetradecanoic	cis-10-Pentodecanoic	Pentadecanoic	Palmitoleic	Hexadecanoic	cis-10-Heptadecenoic	Heptadecanoic	gamma-Linolenic	Linoleic	cis-Oleic	Linoelaidic
Amaranthus retroflexus L.	n.d.	n.d.	n.d.	n.d.	n.d.	0.512± 0.043	n.d.	0.3± 0.026	n.d.	5.342± 0.147	0.291± 0.019	0.314± 0.014	n.d.	27.213± 1.164	6.921± 0.205	1.796± 0.161
Arctium lappa L.	n.d.	n.d.	n.d.	n.d.	n.d.	0.522± 0.024	n.d.	0.308± 0.021	4.945± 0.278	7.893± 0.222	0.33± 0.01	0.323± 0.038	n.d.	98.656± 2.823	45.747± 1.136	24.524± 1.941
Arenaria serpyllifolia agg.	n.d.	n.d.	n.d.	0.4± 0.009	n.d.	0.452± 0.025	n.d.	0.291± 0.016	0.447± 0.047	9.666± 0.562	0.304± 0.022	0.385± 0.019	7.88± 0.209	24.232± 0.776	5.45± 0.235	n.d.
Bidens tripartita L.	n.d.	n.d.	n.d.	n.d.	0.613± 0.006	0.608± 0.045	0.295± 0.008	0.313± 0.023	1.222± 0.058	17.395± 0.478	0.54± 0.052	0.534± 0.068	n.d.	113.045± 2.981	50.948± 0.292	49.219± 2.181
Campanula trachelium L.	n.d.	n.d.	n.d.	n.d.	n.d.	0.559± 0.022	0.309± 0.01	0.324± 0.005	12.367± 0.357	18.169± 0.572	0.332± 0.029	0.448± 0.047	n.d.	165.164± 2.715	20.511± 1.764	75.292± 0.743
Capsella bursa-pastoris (L.) Med.	n.d.	0.4± 0.02	n.d.	0.451± 0.03	0.623± 0.025	1.3± 0.02	n.d.	0.333± 0.014	0.817± 0.02	23.258± 0.88	0.51± 0.142	0.567± 0.065	n.d.	69.437± 0.57	32.964± 0.837	12.787± 0.857
Chenopodium album L.	n.d.	0.353± 0.005	n.d.	0.406± 0.016	n.d.	1.607± 0.042	n.d.	0.703± 0.031	11.102± 0.467	15.07± 0.2	0.338± 0.007	0.87± 0.046	n.d.	128.649± 3.382	57.327± 1.496	57.812± 2.537
Cichorium intybus L.	n.d.	n.d.	n.d.	0.363± 0.015	n.d.	n.d.	0.473± 0.046	0.213± 0.015	0.64± 0.017	4.005± 0.114	0.214± 0.032	0.254± 0.008	0.787± 0.071	21.084± 0.959	24.176± 0.325	6.274± 0.264
Cirsium arvense (L.) Scop.	n.d.	n.d.	n.d.	n.d.	0.83± 0.026	1.29± 0.07	n.d.	0.397± 0.015	n.d.	3.473± 0.115	n.d.	0.523± 0.015	0.773± 0.031	44.39± 2.052	9.853± 0.862	5.51± 0.574
Consolida regalis S.F.Gray	0.302± 0.018	0.33± 0.026	n.d.	0.345± 0.023	n.d.	0.5± 0.009	0.138± 0.106	0.286± 0.031	0.698± 0.016	20.22± 0.486	16.317± 0.477	0.482± 0.016	n.d.	64.937± 2.034	28.767± 1.042	28.722± 1.718
Crepis biennis L.	n.d.	n.d.	n.d.	0.422± 0.026	n.d.	0.566± 0.05	0.221± 0.031	0.28± 0.02	0.673± 0.062	13.479± 0.585	2.481± 0.25	0.299± 0.08	n.d.	57± 1.361	26.781± 2.168	26.023± 1.657
Descurainia sophia (L.) Prantl	n.d.	n.d.	n.d.	0.43± 0.01	n.d.	0.528± 0.023	0.303± 0.012	0.308± 0.011	18.041± 0.864	24.663± 1.436	n.d.	0.827± 0.076	n.d.	237.369± 3.282	118.494± 12.376	52.98± 3.106
Fumaria officinalis L.	n.d.	n.d.	n.d.	0.41± 0.418	n.d.	0.66± 0.667	n.d.	0.31± 0.299	0.63± 0.643	3.14± 3.386	0.32± 0.301	0.32± 0.306	n.d.	3.54± 3.546	12.32± 13.708	4.32± 4.266
Galinsoga parviflora Cav.	n.d.	n.d.	n.d.	n.d.	n.d.	0.471± 0.028	n.d.	0.301± 0.018	0.543± 0.068	5.265± 0.247	0.317± 0.021	0.345± 0.058	n.d.	34.004± 1.89	9.411± 0.169	3.529± 0.292
Galium aparine L.	n.d.	n.d.	n.d.	0.423± 0.025	n.d.	1.053± 0.085	0.68± 0.036	0.737± 0.133	0.64± 0.036	6.814± 0.236	0.284± 0.025	0.657± 0.023	n.d.	89.717± 2.838	40.221± 1.539	37.859± 2.304
Lapsana communis L.	n.d.	n.d.	n.d.	n.d.	n.d.	0.5± 0.035	0.293± 0.021	0.667± 0.015	3.352± 0.085	5.177± 0.092	n.d.	0.359± 0.047	n.d.	31.518± 1.293	8.308± 0.344	3.898± 0.063
Leonurus cardiaca L.	0.33± 0.335	n.d.	n.d.	0.49± 0.471	n.d.	0.76± 0.703	0.24± 0.238	0.27± 0.272	0.62± 0.571	11.85± 12.022	1.86± 1.703	0.5± 0.496	n.d.	148.42± 150.243	66.74± 67.122	19.22± 19.198
Lepidium ruderale L.	n.d.	n.d.	n.d.	0.416± 0.015	n.d.	0.618± 0.03	n.d.	0.335± 0.061	0.463± 0.012	10.81± 0.19	0.31± 0.018	0.557± 0.136	n.d.	34.806± 0.247	16.488± 0.305	15.828± 0.292
Melilotus albus Med.	n.d.	n.d.	n.d.	0.379± 0.019	n.d.	0.434± 0.021	n.d.	0.313± 0.097	4.224± 0.287	6.457± 0.294	n.d.	0.324± 0.005	n.d.	29.913± 1.672	11.013± 0.729	5.636± 0.279
Potentilla argentea L.	n.d.	n.d.	n.d.	0.448± 0.04	n.d.	0.405± 0.027	0.245± 0.023	0.28± 0.02	5.41± 0.276	8.253± 0.11	n.d.	0.396± 0.015	n.d.	55.265± 1.251	25.112± 0.646	24.398± 1.449
Silene latifolia alba (Mill.) Greut. et Burdet	n.d.	n.d.	n.d.	n.d.	n.d.	0.557± 0.097	0.24± 0.026	0.57± 0.01	3.383± 0.146	4.843± 0.175	1.687± 0.031	0.337± 0.04	n.d.	27.085± 0.783	4.731± 0.064	1.42± 0.062
Sisymbrium loeselii L.	n.d.	n.d.	n.d.	n.d.	n.d.	0.746± 0.008	0.299± 0.009	0.57± 0.01	2.473± 0.112	3.307± 0.463	1.687± 0.031	0.337± 0.04	n.d.	24.38± 0.712	6.731± 0.064	1.42± 0.062
Stellaria media (L.) Vill.	n.d.	n.d.	n.d.	0.412± 0.021	n.d.	1.627± 0.059	n.d.	0.295± 0.028	n.d.	7.237± 0.121	n.d.	0.259± 0.038	4.89± 0.208	13.886± 0.558	3.888± 0.263	1.125± 0.1
Taraxacum officinale agg.	n.d.	n.d.	n.d.	n.d.	n.d.	0.528± 0.028	0.23± 0.03	0.295± 0.009	6.446± 0.576	8.474± 0.255	n.d.	0.354± 0.007	5.338± 0.715	60.816± 1.595	28.427± 0.215	25.012± 3.005
Thlapsi arvense L.	n.d.	0.415± 0.008	n.d.	0.498± 0.023	0.293± 0.021	0.577± 0.04	n.d.	0.667± 0.061	0.4± 0.077	6.704± 0.281	n.d.	0.322± 0.004	n.d.	38.634± 1.943	16.879± 1.414	16.523± 2.015
Tripleurospermum inodorum (L.) Schultz-Bip.	n.d.	n.d.	0.243± 0.012	n.d.	n.d.	1.533± 0.051	n.d.	0.276± 0.012	0.677± 0.031	7.162± 0.243	n.d.	0.336± 0.005	n.d.	60.604± 1.682	29.196± 0.792	n.d.
Urtica dioica L.	n.d.	n.d.	n.d.	n.d.	n.d.	1.569± 0.127	n.d.	0.279± 0.028	0.284± 0.04	2.796± 0.186	0.305± 0.005	0.316± 0.029	n.d.	53.398± 2.272	5.597± 0.536	1.49± 0.053
Viola arvensis Murray	0.304± 0.019	n.d.	n.d.	0.348± 0.016	n.d.	1.517± 0.038	n.d.	0.233± 0.005	0.731± 0.045	17.829± 0.427	0.807± 0.023	0.763± 0.015	n.d.	118.455± 2.021	53.832± 1.243	53.074± 1.409

Appendix S1: Table 3. (Continued)

Species	trans-Elaidic	Stearic	cis-5,8,11,14-Eicosatetranoic	cis-5,8,11,14,17-Eicosapentanoic	cis-8,11,14-Eicosatrienoic	cis-11,14-Eicosadienoic	cis-11-Eicosenoic	Eicosanoic	Heneicosanoic	cis-4,7,10,13,16,19-Docosahexaenoic	cis-13,16-Docosadienoic	Erucic	Docosanoic	Tricosanoic	cis-15-Tetracosenoic	Tetracosanoic
Amaranthus retroflexus L.	n.d.	1.388± 0.064	n.d.	n.d.	0.352± 0.019	n.d.	0.76± 0.09	0.748± 0.02	n.d.	n.d.	n.d.	0.425± 0.014	0.611± 0.01	0.347± 0.015	n.d.	n.d.
Arctium lappa L.	n.d.	3.508± 0.246	n.d.	n.d.	0.507± 0.015	0.613± 0.012	0.643± 0.047	1.244± 0.085	0.325± 0.023	n.d.	n.d.	0.743± 0.057	0.917± 0.065	0.355± 0.031	n.d.	0.757± 0.067
Arenaria serpyllifolia agg.	0.955± 0.085	1.506± 0.043	n.d.	n.d.	0.308± 0.014	0.261± 0.01	0.37± 0.026	0.625± 0.013	n.d.	n.d.	n.d.	0.842± 0.039	0.597± 0.014	0.303± 0.015	0.308± 0.023	0.768± 0.064
Bidens tripartita L.	n.d.	1.765± 0.097	n.d.	n.d.	0.432± 0.024	n.d.	0.474± 0.022	1.016± 0.039	n.d.	n.d.	n.d.	0.314± 0.032	1.69± 0.173	0.312± 0.02	n.d.	1.643± 0.106
Campanula trachelium L.	n.d.	5.61± 0.298	n.d.	n.d.	0.321± 0.021	0.626± 0.031	0.349± 0.026	0.738± 0.097	n.d.	n.d.	n.d.	0.874± 0.056	n.d.	n.d.	0.356± 0.023	n.d.
Capsella bursa-pastoris (L.) Med.	15.545± 2.652	15.824± 0.804	n.d.	n.d.	15.393± 0.631	6.185± 0.274	25.251± 2.318	6.248± 0.29	0.273± 0.047	n.d.	n.d.	1.701± 0.153	1.554± 0.144	0.281± 0.051	1.11± 0.03	0.934± 0.066
Chenopodium album L.	n.d.	5.217± 0.166	n.d.	n.d.	0.78± 0.02	0.657± 0.025	0.783± 0.145	4.017± 0.19	0.797± 0.023	n.d.	n.d.	n.d.	1.777± 0.131	0.275± 0.03	n.d.	5.244± 0.093
Cichorium intybus L.	n.d.	1.555± 0.083	n.d.	n.d.	7.785± 0.293	n.d.	13.954± 0.427	0.558± 0.051	n.d.	n.d.	1.337± 0.126	0.235± 0.04	0.49± 0.046	n.d.	n.d.	0.467± 0.061
Cirsium arvense (L.) Scop.	2.173± 0.195	3.213± 0.284	n.d.	n.d.	0.583± 0.045	n.d.	0.8± 0.04	0.487± 0.061	0.347± 0.038	0.427± 0.025	1.49± 0.026	0.42± 0.017	0.44± 0.087	0.397± 0.042	n.d.	0.367± 0.023
Consolida regalis S.F.Gray	2.092± 0.102	5.879± 0.312	n.d.	n.d.	11.677± 0.52	3.832± 0.272	19.405± 0.69	4.273± 0.295	0.257± 0.04	n.d.	1.578± 0.076	23.069± 2.772	1.772± 0.093	0.277± 0.052	2.507± 0.13	1.174± 0.116
Crepis biennis L.	15.122± 0.479	3.496± 0.546	n.d.	0.298± 0.028	0.272± 0.029	0.283± 0.03	0.837± 0.085	0.843± 0.065	n.d.	0.336± 0.033	n.d.	1.347± 0.095	0.591± 0.069	n.d.	0.378± 0.054	n.d.
Descurainia sophia (L.) Prantl	n.d.	7.23± 0.284	n.d.	n.d.	0.358± 0.013	0.316± 0.007	0.435± 0.038	1.161± 0.105	0.307± 0.011	n.d.	n.d.	n.d.	2.485± 0.152	0.324± 0.027	n.d.	1.193± 0.046
Fumaria officinalis L.	n.d.	1.24± 1.085	n.d.	n.d.	0.25± 0.303	0.24± 0.243	0.65± 0.647	0.62± 0.605	0.28± 0.282	n.d.	n.d.	1.29± 1.297	0.76± 0.767	n.d.	n.d.	0.65± 0.625
Galinsoga parviflora Cav.	n.d.	1.403± 0.284	n.d.	n.d.	0.564± 0.074	0.376± 0.029	0.7± 0.019	1.911± 0.116	0.311± 0.001	n.d.	n.d.	1.331± 0.042	1.891± 0.046	n.d.	1.08± 0.207	1.713± 0.047
Galium aparine L.	n.d.	4.025± 0.157	n.d.	n.d.	0.349± 0.026	n.d.	0.623± 0.006	1.723± 0.172	0.293± 0.011	n.d.	n.d.	n.d.	n.d.	0.303± 0.006	n.d.	1.603± 0.068
Lapsana communis L.	n.d.	2.303± 0.042	0.307± 0.006	0.309± 0.027	n.d.	n.d.	0.431± 0.095	1.066± 0.087	0.282± 0.014	0.316± 0.01	n.d.	n.d.	0.746± 0.027	n.d.	n.d.	0.612± 0.014
Leonurus cardiaca L.	n.d.	9.47± 9.685	n.d.	n.d.	1.32± 1.372	1.6± 1.609	n.d.	1.65± 1.697	n.d.	n.d.	n.d.	1.18± 1.223	1.76± 1.812	1.43± 1.453	1.76± 1.794	2.63± 2.531
Lepidium ruderale L.	n.d.	4.533± 0.246	n.d.	n.d.	5.643± 0.235	2.369± 0.236	8.356± 0.084	2.039± 0.138	n.d.	n.d.	n.d.	1.266± 0.207	0.611± 0.149	0.437± 0.084	0.863± 0.125	0.6± 0.172
Melilotus albus Med.	n.d.	1.515± 0.071	n.d.	n.d.	0.303± 0.006	0.33± 0.009	0.308± 0.033	1.677± 0.045	0.302± 0.014	n.d.	n.d.	n.d.	0.78± 0.02	0.315± 0.018	n.d.	0.594± 0.054
Potentilla argentea L.	n.d.	3.065± 0.094	n.d.	n.d.	n.d.	0.363± 0.029	0.495± 0.032	2.314± 0.177	n.d.	n.d.	n.d.	0.276± 0.025	2.009± 0.136	0.306± 0.011	n.d.	1.776± 0.07
Silene latifolia alba (Mill.) Greut. et Burdet	n.d.	1.142± 0.183	n.d.	n.d.	0.303± 0.006	0.733± 0.129	0.723± 0.038	0.645± 0.043	n.d.	n.d.	n.d.	n.d.	0.841± 0.244	0.386± 0.062	n.d.	0.513± 0.094
Sisymbrium loeselii L.	n.d.	1.142± 0.183	n.d.	n.d.	0.618± 0.037	0.733± 0.129	0.822± 0.035	0.645± 0.043	n.d.	n.d.	n.d.	n.d.	0.841± 0.244	0.386± 0.062	n.d.	0.513± 0.094
Stellaria media (L.) Vill.	n.d.	1.326± 0.061	n.d.	n.d.	0.331± 0.011	0.68± 0.104	0.803± 0.1	0.639± 0.037	n.d.	0.327± 0.03	n.d.	0.917± 0.025	0.61± 0.041	0.338± 0.036	n.d.	0.612± 0.003
Taraxacum officinale agg.	n.d.	3.426± 0.36	n.d.	n.d.	0.473± 0.025	n.d.	0.469± 0.04	1.787± 0.179	0.32± 0.026	n.d.	n.d.	n.d.	1.788± 0.121	0.342± 0.007	0.394± 0.01	n.d.
Thlapsi arvense L.	n.d.	1.481± 0.126	n.d.	n.d.	8.432± 0.88	4.121± 0.198	14.946± 0.642	1.092± 0.132	n.d.	n.d.	58.753± 1.39	64.2± 3.304	0.745± 0.043	0.31± 0.018	5.261± 0.539	0.618± 0.077
Tripleurospermum inodorum (L.) Schultz-Bip.	n.d.	2.88± 0.176	n.d.	n.d.	0.371± 0.018	0.321± 0.018	0.637± 0.061	1.567± 0.047	0.302± 0.007	n.d.	n.d.	n.d.	1.28± 0.061	n.d.	0.348± 0.017	n.d.
Urtica dioica L.	n.d.	0.999± 0.064	n.d.	n.d.	0.825± 0.052	0.791± 0.037	0.402± 0.013	2.691± 0.136	n.d.	n.d.	n.d.	0.788± 0.008	1.695± 0.135	n.d.	n.d.	1.635± 0.026
Viola arvensis Murray	n.d.	3.1± 0.174	n.d.	n.d.	0.323± 0.012	0.697± 0.015	0.516± 0.031	1.493± 0.04	n.d.	n.d.	n.d.	n.d.	1.617± 0.035	n.d.	n.d.	0.605± 0.018

Appendix S1: Table 4. The amount of fatty acids found on the seed surface. The fatty acids were isolated and derivatized into corresponding volatile methyl esters and then quantified via GC-MS method. The highest content of all surface fatty acids was found on the seeds of *C. arvense* (114.12±3.21 mg/g_{DW}). Other species had significantly lower content of surface fatty acids. The lowest amount of all surface fatty acids (6.07±0.32 mg/g_{DW}) was found on the seeds of *A. retroflexus.*

Species	Butanoic	Hexanoic	Octanoic	Decanoic	Undecanoic	Dodecanoic	Tridecanoic	Myristoleic	Tetradecanoic	cis-10-Pentodecanoic	Pentadecanoic	Palmitoleic	Hexadecanoic	cis-10-Heptadecenoic	Heptadecanoic	gamma - Linolenic	Linoleic	cis-Oleic	Linolealidic
Amaranthus retroflexus L.	n.d.	n.d.	n.d.	n.d.	n.d.	n.d.	n.d.	n.d.	n.d.	n.d.	n.d.	n.d.	1.591± 0.063	n.d.	n.d.	n.d.	0.771± 0.093	1.166± 0.077	n.d.
Arctium lappa L.	n.d.	n.d.	n.d.	n.d.	n.d.	n.d.	n.d.	n.d.	n.d.	n.d.	0.58± 0.034	n.d.	1.49± 0.04	n.d.	n.d.	n.d.	0.672± 0.026	1.219± 0.126	0.537± 0.098
Arenaria serpyllifolia agg.	n.d.	n.d.	n.d.	n.d.	n.d.	n.d.	n.d.	n.d.	n.d.	n.d.	0.612± 0.01	n.d.	1.589± 0.03	n.d.	n.d.	n.d.	0.698± 0.042	1.201± 0.103	n.d.
Bidens tripartita L.	n.d.	n.d.	n.d.	n.d.	n.d.	n.d.	n.d.	0.603± 0.025	n.d.	n.d.	0.34± 0.01	n.d.	1.751± 0.082	n.d.	n.d.	n.d.	1.038± 0.078	1.366± 0.166	0.607± 0.03
Campanula trachelium L.	n.d.	n.d.	n.d.	n.d.	n.d.	n.d.	n.d.	n.d.	0.493± 0.042	n.d.	n.d.	n.d.	1.613± 0.035	n.d.	n.d.	n.d.	1.592± 0.145	1.286± 0.108	0.643± 0.091
Capsella bursa-pastoris (L.) Med.	n.d.	n.d.	n.d.	n.d.	n.d.	n.d.	n.d.	0.676± 0.042	1.078± 0.05	n.d.	n.d.	0.645± 0.048	1.611± 0.146	0.595± 0.037	n.d.	n.d.	1.157± 0.096	1.362± 0.049	n.d.
Chenopodium album L.	n.d.	n.d.	n.d.	n.d.	n.d.	n.d.	n.d.	n.d.	1.261± 0.102	n.d.	0.614± 0.043	9.89± 0.656	9.04± 0.338	n.d.	0.801± 0.093	n.d.	45.833± 1.652	23.635± 1.118	8.057± 0.633
Cichorium intybus L.	n.d.	n.d.	n.d.	n.d.	n.d.	n.d.	n.d.	n.d.	n.d.	0.604± 0.014	n.d.	0.591± 0.01	1.615± 0.039	n.d.	n.d.	0.672± 0.025	0.649± 0.019	1.2± 0.046	n.d.
Cirsium arvense (L.) Scop.	n.d.	n.d.	n.d.	n.d.	n.d.	n.d.	n.d.	0.57± 0.044	1.137± 0.095	n.d.	n.d.	n.d.	1.351± 0.529	n.d.	n.d.	0.694± 0.01	0.699± 0.019	1.202± 0.089	0.5± 0.02
Consolida regalis S.F.Gray	n.d.	n.d.	n.d.	n.d.	n.d.	n.d.	n.d.	n.d.	n.d.	n.d.	n.d.	n.d.	0.621± 0.026	1.637± 0.029	n.d.	n.d.	0.639± 0.054	0.782± 0.069	1.22± 0.095
Crepis biennis L.	n.d.	n.d.	n.d.	n.d.	n.d.	n.d.	n.d.	n.d.	0.556± 0.079	0.077± 0.006	0.233± 0.015	0.588± 0.105	2.831± 0.172	2.495± 0.23	0.22± 0.017	n.d.	n.d.	22.943± 1.374	15.641± 1.644
Descurainia sophia (L.) Prantl	n.d.	n.d.	n.d.	n.d.	n.d.	n.d.	n.d.	n.d.	n.d.	n.d.	n.d.	n.d.	2.459± 0.087	n.d.	0.707± 0.006	n.d.	8.825± 0.266	1.796± 0.304	0.758± 0.113
Fumaria officinalis L.	n.d.	n.d.	n.d.	n.d.	n.d.	n.d.	n.d.	n.d.	0.607± 0.012	n.d.	n.d.	0.561± 0.037	0.591± 0.012	1.566± 0.03	0.611± 0.012	n.d.	n.d.	0.701± 0.018	1.218± 0.1
Galinsoga parviflora Cav.	n.d.	n.d.	n.d.	n.d.	n.d.	n.d.	n.d.	n.d.	n.d.	n.d.	n.d.	n.d.	1.797± 0.162	n.d.	n.d.	n.d.	1.022± 0.085	1.29± 0.036	0.567± 0.031
Galium aparine L.	n.d.	n.d.	n.d.	n.d.	n.d.	n.d.	n.d.	n.d.	0.898± 0.076	0.633± 0.048	0.596± 0.016	0.637± 0.021	2.147± 0.065	n.d.	0.612± 0.003	n.d.	13.788± 0.958	2.445± 0.258	0.804± 0.075
Lapsana communis L.	n.d.	n.d.	n.d.	n.d.	n.d.	n.d.	n.d.	n.d.	n.d.	n.d.	0.594± 0.007	n.d.	1.601± 0.02	n.d.	n.d.	n.d.	0.801± 0.05	1.076± 0.116	0.517± 0.02
Leonurus cardiaca L.	n.d.	n.d.	n.d.	n.d.	n.d.	n.d.	n.d.	n.d.	0.66± 0.61	n.d.	n.d.	0.58± 0.585	0.61± 0.602	1.54± 1.576	n.d.	n.d.	n.d.	2.56± 2.817	1.77± 1.862
Lepidium ruderale L.	n.d.	n.d.	n.d.	n.d.	n.d.	n.d.	n.d.	n.d.	n.d.	n.d.	0.315± 0.015	n.d.	0.733± 0.038	n.d.	0.572± 0.057	n.d.	2.462± 0.278	1.347± 0.101	1.929± 0.146
Melilotus albus Med.	n.d.	n.d.	n.d.	n.d.	n.d.	n.d.	n.d.	n.d.	n.d.	n.d.	n.d.	n.d.	1.662± 0.073	n.d.	n.d.	n.d.	0.764± 0.049	1.187± 0.068	n.d.
Potentilla argentea L.	n.d.	n.d.	n.d.	n.d.	n.d.	n.d.	n.d.	n.d.	n.d.	n.d.	n.d.	n.d.	1.711± 0.065	n.d.	n.d.	n.d.	1.18± 0.04	1.569± 0.114	0.659± 0.031
Silene latifolia alba (Mill.) Greut. et Burdet	n.d.	n.d.	n.d.	n.d.	n.d.	n.d.	n.d.	n.d.	0.563± 0.015	n.d.	0.54± 0.041	n.d.	n.d.	1.478± 0.111	n.d.	n.d.	0.685± 0.033	0.66± 0.061	n.d.
Sisymbrium loeselii L.	n.d.	n.d.	n.d.	n.d.	n.d.	n.d.	n.d.	n.d.	0.674± 0.021	n.d.	0.54± 0.041	n.d.	n.d.	2.478± 0.111	n.d.	n.d.	0.951± 0.03	0.66± 0.061	2.199± 0.105
Stellaria media (L.) Vill.	n.d.	n.d.	n.d.	n.d.	n.d.	n.d.	n.d.	0.613± 0.006	1.157± 0.145	n.d.	n.d.	n.d.	1.587± 0.09	n.d.	n.d.	0.736± 0.065	0.795± 0.048	1.381± 0.153	0.565± 0.092
Taraxacum officinale agg.	n.d.	n.d.	n.d.	n.d.	n.d.	n.d.	n.d.	n.d.	n.d.	n.d.	n.d.	0.711± 0.074	1.407± 0.271	n.d.	n.d.	n.d.	1.421± 0.333	1.4± 0.044	0.598± 0.076
Thlapsi arvense L.	n.d.	n.d.	n.d.	n.d.	n.d.	n.d.	n.d.	n.d.	n.d.	n.d.	0.597± 0.005	n.d.	1.49± 0.099	n.d.	n.d.	n.d.	0.719± 0.046	n.d.	0.48± 0.046
Tripleurospermum inodorum (L.) Schu.-Bip.	n.d.	n.d.	n.d.	n.d.	n.d.	n.d.	n.d.	n.d.	1.081± 0.062	n.d.	n.d.	0.617± 0.016	1.638± 0.17	n.d.	n.d.	n.d.	1.245± 0.073	1.409± 0.175	n.d.
Urtica dioica L.	n.d.	n.d.	n.d.	n.d.	n.d.	n.d.	n.d.	n.d.	1.339± 0.287	n.d.	n.d.	n.d.	2.511± 0.171	n.d.	n.d.	n.d.	22.325± 0.813	3.264± 0.265	1.065± 0.075
Viola arvensis Murray	n.d.	n.d.	n.d.	n.d.	n.d.	n.d.	n.d.	n.d.	1.091± 0.036	n.d.	n.d.	n.d.	6.255± 0.083	0.707± 0.035	0.739± 0.035	n.d.	6.079± 0.201	4.59± 0.131	1.727± 0.129

Appendix S1: Table 4. (Continued)

Species	trans-Elaidic	Stearic	cis-5,8,11,14-Eicosatetranoic	cis-5,8,11,14,17-Eicosapentanoic	cis-8,11,14-Eicosatrienoic	cis-11,14-Eicosadienoic	cis-11-Eicosenoic	Eicosanoic	Heneicosanoic	cis-4,7,10,13,16,19-Docosahexaenoic	cis-13,16-Docasadienoic	Erucic	Docosanoic	Tricosanoic	cis-15-Tetracosenoic	Tetracosanoic
Amaranthus retroflexus L.	n.d.	1.165± 0.091	n.d.	n.d.	n.d.	n.d.	0.632± 0.025	n.d.	n.d.	n.d.	n.d.	0.741± 0.03	n.d.	n.d.	n.d.	n.d.
Arctium lappa L.	n.d.	1.184± 0.054	n.d.	n.d.	n.d.	0.599± 0.028	0.556± 0.01	n.d.	n.d.	n.d.	n.d.	0.716± 0.027	n.d.	n.d.	n.d.	n.d.
Arenaria serpyllifolia agg.	n.d.	1.113± 0.049	n.d.	n.d.	n.d.	n.d.	0.599± 0.018	n.d.	n.d.	n.d.	n.d.	0.751± 0.044	n.d.	n.d.	n.d.	n.d.
Bidens tripartita L.	n.d.	1.287± 0.068	n.d.	n.d.	n.d.	n.d.	0.546± 0.095	n.d.	n.d.	n.d.	n.d.	0.255± 0.005	1.406± 0.117	n.d.	n.d.	1.41± 0.061
Campanula trachelium L.	n.d.	1.272± 0.052	n.d.	n.d.	n.d.	0.6± 0.01	n.d.	n.d.	n.d.	n.d.	n.d.	0.674± 0.031	n.d.	n.d.	n.d.	n.d.
Capsella bursa-pastoris (L.) Med.	n.d.	1.348± 0.011	n.d.	n.d.	0.772± 0.023	0.62± 0.03	0.622± 0.039	1.292± 0.024	n.d.	n.d.	n.d.	0.77± 0.044	1.23± 0.05	n.d.	n.d.	n.d.
Chenopodium album L.	n.d.	5.107± 0.117	n.d.	n.d.	0.721± 0.063	0.609± 0.008	0.734± 0.041	3.208± 0.109	0.706± 0.005	n.d.	n.d.	n.d.	1.687± 0.083	n.d.	0.811± 0.072	1.404± 0.048
Cichorium intybus L.	n.d.	1.252± 0.063	n.d.	n.d.	n.d.	n.d.	0.577± 0.025	n.d.	n.d.	n.d.	1.185± 0.117	0.719± 0.076	n.d.	n.d.	n.d.	n.d.
Cirsium arvense (L.) Scop	n.d.	1.306± 0.043	n.d.	n.d.	n.d.	n.d.	0.585± 0.031	1.202± 0.083	n.d.	n.d.	1.163± 0.045	0.691± 0.047	1.134± 0.162	n.d.	n.d.	1.291± 0.057
Consolida regalis S.F.Gray	n.d.	1.334± 0.179	n.d.	n.d.	n.d.	n.d.	0.613± 0.04	0.603± 0.014	n.d.	n.d.	n.d.	1.155± 0.075	0.653± 0.084	n.d.	n.d.	n.d.
Crepis biennis L.	4.536± 0.377	2.529± 0.215	n.d.	0.19± 0.02	0.213± 0.021	n.d.	0.576± 0.056	0.612± 0.04	n.d.	n.d.	n.d.	1.072± 0.117	0.597± 0.152	n.d.	n.d.	n.d.
Descurainia sophia (L.) Prantl	n.d.	1.585± 0.056	n.d.	n.d.	n.d.	0.652± 0.007	n.d.	1.257± 0.127	n.d.	n.d.	n.d.	n.d.	1.517± 0.025	n.d.	n.d.	n.d.
Fumaria officinalis L.	0.496± 0.015	n.d.	n.d.	n.d.	n.d.	n.d.	0.624± 0.034	0.586± 0.02	n.d.	n.d.	n.d.	1.27± 0.158	0.683± 0.035	n.d.	n.d.	n.d.
Galinsoga parviflora Cav.	n.d.	1.198± 0.152	n.d.	n.d.	n.d.	n.d.	n.d.	1.243± 0.093	n.d.	n.d.	n.d.	0.733± 0.035	1.297± 0.032	n.d.	0.909± 0.038	1.347± 0.095
Galium aparine L.	n.d.	1.705± 0.031	n.d.	n.d.	n.d.	n.d.	0.605± 0.018	1.213± 0.064	n.d.	n.d.	n.d.	n.d.	n.d.	n.d.	n.d.	1.441± 0.182
Lapsana communis L.	n.d.	1.199± 0.063	n.d.	n.d.	n.d.	n.d.	n.d.	n.d.	n.d.	n.d.	n.d.	n.d.	0.62± 0.03	n.d.	n.d.	n.d.
Leonurus cardiaca L.	n.d.	1.52± 1.451	n.d.	n.d.	n.d.	0.7± 0.722	0.6± 0.604	0.55± 0.571	1.19± 1.179	n.d.	n.d.	1.1± 1.083	0.68± 0.671	1.25± 1.224	n.d.	n.d.
Lepidium ruderale L.	n.d.	1.008± 0.076	n.d.	n.d.	n.d.	0.687± 0.055	0.714± 0.07	0.627± 0.016	n.d.	n.d.	n.d.	1.112± 0.089	0.35± 0.036	0.28± 0.01	n.d.	n.d.
Melilotus albus Med.	n.d.	1.257± 0.056	n.d.	n.d.	n.d.	n.d.	n.d.	1.181± 0.057	n.d.	n.d.	n.d.	n.d.	0.727± 0.04	n.d.	n.d.	n.d.
Potentilla argentea L.	n.d.	1.245± 0.052	n.d.	n.d.	n.d.	0.367± 0.032	n.d.	1.393± 0.225	n.d.	n.d.	n.d.	n.d.	1.456± 0.095	n.d.	n.d.	1.436± 0.251
Silene latifolia alba (Mill.) Greut. et Burdet	n.d.	1.194± 0.04	n.d.	n.d.	n.d.	0.713± 0.006	0.631± 0.036	0.529± 0.055	n.d.	n.d.	n.d.	n.d.	0.718± 0.029	n.d.	n.d.	n.d.
Sisymbrium loeselii L.	n.d.	1.194± 0.04	n.d.	n.d.	n.d.	0.713± 0.006	0.631± 0.036	0.4± 0.023	n.d.	n.d.	n.d.	n.d.	0.718± 0.029	n.d.	n.d.	n.d.
Stellaria media (L.) Vill.	n.d.	1.186± 0.032	n.d.	n.d.	n.d.	0.58± 0.034	0.647± 0.025	n.d.	n.d.	n.d.	n.d.	0.751± 0.019	n.d.	n.d.	n.d.	n.d.
Taraxacum officinale agg.	n.d.	1.233± 0.09	n.d.	n.d.	n.d.	n.d.	n.d.	0.6± 0.02	n.d.	n.d.	n.d.	n.d.	1.415± 0.196	n.d.	0.27± 0.026	n.d.
Thlapsi arvense L.	n.d.	1.131± 0.057	n.d.	n.d.	n.d.	0.658± 0.037	n.d.	n.d.	n.d.	n.d.	1.343± 0.205	0.766± 0.068	n.d.	n.d.	n.d.	n.d.
Tripleurospermum inodorum (L.) Schu.-Bip.	n.d.	1.285± 0.057	n.d.	n.d.	n.d.	n.d.	0.592± 0.044	1.185± 0.135	n.d.	n.d.	n.d.	n.d.	1.279± 0.06	n.d.	n.d.	n.d.
Urtica dioica L.	n.d.	0.65± 0.052	n.d.	n.d.	0.74± 0.036	0.686± 0.012	n.d.	1.587± 0.055	0.732± 0.014	n.d.	n.d.	0.732± 0.02	1.584± 0.177	n.d.	n.d.	1.522± 0.124
Viola arvensis Murray	n.d.	2.453± 0.126	n.d.	n.d.	n.d.	0.605± 0.062	n.d.	1.274± 0.041	n.d.	n.d.	n.d.	n.d.	1.302± 0.065	n.d.	n.d.	n.d.

Appendix S1: Table 5. Volatiles from imbibed seeds. Volatile compounds released by seeds were after imbibition and consecutive incubation (24 hours in 25 °C.) into headspaces vials and detected by using common GCMS platform. The highest amount of volatile compound was found in seeds of *Sisymbrium loeselii* L. (4.83% of determinated volatiles) and lowest in seeds of *Amaranthus retroflexus* L. (0.03 %).

**Species**	Acetaldehyde	Acetoni0ile	Ethanol	Ethanthiol	Ethyl acetate	Chloroform	Methanol	Methylene chloride	n-Hexane	Propanol	Pyruvic acid	tert-Butanol
Amaranthus retroflexus L.	n.d.	0.03	n.d.	n.d.	n.d.	n.d.	n.d.	n.d.	n.d.	n.d.	n.d.	n.d.
Arctium lappa L.	n.d.	0.05	n.d.	0.12	n.d.	0.05	n.d.	n.d.	n.d.	n.d.	n.d.	n.d.
Arenaria serpyllifolia agg.	n.d.	0.76	1.81	n.d.	n.d.	0.05	0.31	0.09	n.d.	n.d.	n.d.	n.d.
Bidens tripartita L.	n.d.	0.24	n.d.	n.d.	n.d.	0.09	n.d.	0.11	n.d.	n.d.	n.d.	n.d.
Campanula trachelium L.	n.d.	0.06	0.04	n.d.	n.d.	n.d.	n.d.	n.d.	n.d.	n.d.	n.d.	n.d.
Capsella bursa-pastoris (L.) Med.	n.d.	0.05	1.01	0.35	n.d.	n.d.	n.d.	n.d.	n.d.	0.14	n.d.	n.d.
Chenopodium album L.	n.d.	0.31	0.05	n.d.	n.d.	0.62	n.d.	n.d.	n.d.	n.d.	n.d.	0.05
Cichorium intybus L.	0.23	0.35	1.05	0.16	n.d.	0.17	n.d.	0.08	n.d.	n.d.	n.d.	n.d.
Cirsium arvense (L.) Scop.	n.d.	0.38	2.4	n.d.	n.d.	n.d.	0.23	0.06	n.d.	n.d.	n.d.	n.d.
Consolida regalis S.F.Gray	n.d.	0.17	0.67	n.d.	n.d.	n.d.	n.d.	0.03	n.d.	n.d.	n.d.	n.d.
Crepis biennis L.	n.d.	n.d.	0.83	0.04	n.d.	n.d.	n.d.	n.d.	n.d.	n.d.	n.d.	n.d.
Descurainia sophia (L.) Prantl	n.d.	0.32	n.d.	0.26	n.d.	0.34	n.d.	0.11	n.d.	n.d.	n.d.	n.d.
Fumaria officinalis L.	n.d.	0.3	0.74	0.23	n.d.	n.d.	n.d.	n.d.	n.d.	n.d.	n.d.	n.d.
Galinsoga parviflora Cav.	n.d.	0.2	0.05	n.d.	n.d.	n.d.	n.d.	n.d.	n.d.	n.d.	n.d.	n.d.
Galium aparine L.	n.d.	n.d.	0.16	n.d.	n.d.	0.04	n.d.	n.d.	n.d.	n.d.	n.d.	n.d.
Lapsana communis L.	n.d.	0.07	0.18	0.41	n.d.	0.03	n.d.	n.d.	n.d.	n.d.	n.d.	n.d.
Leonurus cardiaca L.	n.d.	0.33	n.d.	0.07	n.d.	0.07	n.d.	0.07	n.d.	n.d.	n.d.	n.d.
Lepidium ruderale L.	n.d.	0.1	0.2	n.d.	n.d.	0.07	n.d.	0.07	n.d.	n.d.	n.d.	n.d.
Melilotus albus Med.	n.d.	n.d.	0.77	n.d.	n.d.	n.d.	n.d.	n.d.	n.d.	n.d.	n.d.	n.d.
Potentilla argentea L.	n.d.	0.1	n.d.	n.d.	n.d.	0.21	n.d.	0.15	n.d.	n.d.	n.d.	n.d.
Silene latifolia alba (Mill.) Greut. et Burdet	n.d.	0.41	1.8	n.d.	n.d.	0.28	n.d.	0.09	n.d.	n.d.	n.d.	n.d.
Sisymbrium loeselii L.	n.d.	1.02	0.35	n.d.	n.d.	0.04	0.11	n.d.	0.13	n.d.	n.d.	3.18
Stellaria media (L.) Vill.	n.d.	0.46	2.71	0.01	n.d.	0.27	n.d.	n.d.	n.d.	n.d.	n.d.	n.d.
Taraxacum officinale agg.	n.d.	0.29	1.42	0.27	n.d.	0.23	n.d.	0.06	n.d.	n.d.	n.d.	n.d.
Thlapsi arvense L.	n.d.	0.07	0.07	0.07	n.d.	n.d.	n.d.	n.d.	n.d.	n.d.	n.d.	n.d.
Tripleurospermum inodorum (L.) Schu.-Bip.	n.d.	0.3	0.19	n.d.	n.d.	0.04	n.d.	0.04	n.d.	n.d.	n.d.	n.d.
Urtica dioica L.	n.d.	0.66	0.34	0.15	n.d.	0.12	n.d.	0.07	n.d.	n.d.	n.d.	n.d.
Viola arvensis Murray	n.d.	0.29	2.44	n.d.	n.d.	0.21	0.21	0.13	n.d.	n.d.	0.03	n.d.

Appendix S1: Table 6. Volatile compounds released from dry seeds into headspaces vials and detected by using common GCMS platform. The highest amount of volatile compounds were found in seeds of *Sisymbrium loeselii* L. (4.8 % of determinated volatiles) and the lowest in seeds of *Amaranthus retroflexus* L., where any volatiles compounds were detected.

Species	Acetaldehyde	Acetoni0ile	Ethanol	Ethanthiol	Chloroform	Methanol	Methylene chloride	n-Hexane	Propanol	Pyruvic acid	tert-Butanol
Amaranthus retroflexus L.	n.d.	n.d.	n.d.	n.d.	n.d.	n.d.	n.d.	n.d.	n.d.	n.d.	n.d.
Arctium lappa L.	n.d.	0.1	n.d.	0.1	0.1	n.d.	n.d.	n.d.	n.d.	n.d.	n.d.
Arenaria serpyllifolia agg.	n.d.	0.8	1.8	n.d.	0.1	0.3	0.1	n.d.	n.d.	n.d.	n.d.
Bidens tripartita L.	n.d.	0.2	n.d.	n.d.	0.1	n.d.	0.1	n.d.	n.d.	n.d.	n.d.
Campanula trachelium L.	n.d.	0.1	n.d.	n.d.	n.d.	n.d.	n.d.	n.d.	n.d.	n.d.	n.d.
Capsella bursa-pastoris (L.) Med.	n.d.	0.1	1	0.4	n.d.	n.d.	n.d.	n.d.	0.1	n.d.	n.d.
Chenopodium album L.	n.d.	0.3	0.1	n.d.	0.6	n.d.	n.d.	n.d.	n.d.	n.d.	0.1
Cichorium intybus L.	0.2	0.4	1.1	0.2	0.2	n.d.	0.1	n.d.	n.d.	n.d.	n.d.
Cirsium arvense (L.) Scop.	n.d.	0.4	2.4	n.d.	n.d.	0.2	0.1	n.d.	n.d.	n.d.	n.d.
Consolida regalis S.F.Gray	n.d.	0.2	0.7	n.d.	n.d.	n.d.	n.d.	n.d.	n.d.	n.d.	n.d.
Crepis biennis L.	n.d.	n.d.	0.8	n.d.	n.d.	n.d.	n.d.	n.d.	n.d.	n.d.	n.d.
Descurainia sophia (L.) Prantl	n.d.	0.3	n.d.	0.3	0.3	n.d.	0.1	n.d.	n.d.	n.d.	n.d.
Fumaria officinalis L.	n.d.	0.3	0.7	0.2	n.d.	n.d.	n.d.	n.d.	n.d.	n.d.	n.d.
Galinsoga parviflora Cav.	n.d.	0.2	0.1	n.d.	n.d.	n.d.	n.d.	n.d.	n.d.	n.d.	n.d.
Galium aparine L.	n.d.	n.d.	0.2	n.d.	n.d.	n.d.	n.d.	n.d.	n.d.	n.d.	n.d.
Lapsana communis L.	n.d.	0.1	0.2	0.4	n.d.	n.d.	n.d.	n.d.	n.d.	n.d.	n.d.
Leonurus cardiaca L.	n.d.	0.3	n.d.	0.1	0.1	n.d.	0.1	n.d.	n.d.	n.d.	n.d.
Lepidium ruderale L.	n.d.	0.1	0.2	n.d.	0.1	n.d.	0.1	n.d.	n.d.	n.d.	n.d.
Melilotus albus Med.	n.d.	n.d.	0.8	n.d.	n.d.	n.d.	n.d.	n.d.	n.d.	n.d.	n.d.
Potentilla argentea L.	n.d.	0.1	n.d.	n.d.	0.2	n.d.	0.2	n.d.	n.d.	n.d.	n.d.
Silene latifolia alba (Mill.) Greut. et Burdet	n.d.	0.4	1.8	n.d.	0.3	n.d.	0.1	n.d.	n.d.	n.d.	n.d.
Sisymbrium loeselii L.	n.d.	1	0.4	n.d.	n.d.	0.1	n.d.	0.1	n.d.	n.d.	3.2
Stellaria media (L.) Vill.	n.d.	0.5	2.7	n.d.	0.3	n.d.	n.d.	n.d.	n.d.	n.d.	n.d.
Taraxacum officinale agg.	n.d.	0.3	1.4	0.3	0.2	n.d.	0.1	n.d.	n.d.	n.d.	n.d.
Thlapsi arvense L.	n.d.	0.1	0.1	0.1	n.d.	n.d.	n.d.	n.d.	n.d.	n.d.	n.d.
Tripleurospermum inodorum (L.) Schultz-Bip.	n.d.	0.3	0.2	n.d.	n.d.	n.d.	n.d.	n.d.	n.d.	n.d.	n.d.
Urtica dioica L.	n.d.	0.7	0.3	0.2	0.1	n.d.	0.1	n.d.	n.d.	n.d.	n.d.
Viola arvensis Murray	n.d.	0.3	2.4	n.d.	0.2	0.2	0.1	n.d.	n.d.	n.d.	n.d.

Appendix S1: Table 7. Other surface compounds. Other surface compounds were isolated from seeds by chloroform. After filtration, chloroform was evaporated under reduced pressure and isolated compounds were dissolved into n-hexane containing 0.1% n-undecane as internal standard for normalization of chromatographic conditions. The results were presented as percentage content of chloroform soluble surface compounds. The majority of surface compounds detected were long-chain alkanes, or their branched counterparts, with significant amounts of phytosterols, such as γ--sitosterol. The composition the surface compounds also varied between the species.

Species	n-Hexadecanoic acid	Ethyl linoleate	Linoleic acid	n-Tricosane	Butyl-9,12-octadecadienoate	n-Hexacosane	1,9-n-Octylhexacosane	n-Octacosane	α-Glyceryl linoleate	n-Triacontane	n-Dotriacontane	Sterol	γ-sitosterol	1,2,4-Dimethoxyestra-1(10),2,4-triene-3,17-diol	Caprin	b-Sitosterol	11-n-Decyltetracosane	Rubrosterone	Trilinolein
Amaranthus retroflexus L.	n.d.	n.d.	n.d.	n.d.	n.d.	n.d.	n.d.	n.d.	10n.d.	n.d.	n.d.	n.d.	n.d.	n.d.	n.d.	n.d.	n.d.	n.d.	n.d.
Arctium lappa L.	n.d.	n.d.	n.d.	n.d.	n.d.	n.d.	n.d.	tr	n.d.	56.89± 1.46	43.07± 1.4	n.d.	n.d.	n.d.	n.d.	n.d.	n.d.	n.d.	n.d.
Arenaria serpyllifolia agg.	n.d.	n.d.	n.d.	tr	0.14± 0.07	n.d.	7.85± 0.33	23.62± 1.36	n.d.	n.d.	n.d.	n.d.	34.11± 0.49	n.d.	n.d.	n.d.	33.19± 0.51	n.d.	n.d.
Bidens tripartita L.	n.d.	n.d.	n.d.	n.d.	n.d.	n.d.	n.d.	tr	n.d.	10n.d.	n.d.	n.d.	n.d.	n.d.	n.d.	n.d.	n.d.	n.d.	n.d.
Campanula trachelium L.	n.d.	n.d.	n.d.	n.d.	65.98± 0.52	n.d.	n.d.	n.d.	n.d.	33.99± 0.54	n.d.	n.d.	n.d.	n.d.	n.d.	n.d.	n.d.	n.d.	n.d.
Capsella bursa-pastoris (L.) Med.	n.d.	n.d.	n.d.	n.d.	n.d.	n.d.	n.d.	tr	n.d.	61.66± 1.23	n.d.	n.d.	n.d.	n.d.	n.d.	n.d.	n.d.	n.d.	n.d.
Chenopodium album L.	n.d.	n.d.	1.24± 0.09	n.d.	5.91± 0.7	n.d.	0.71± 0.14	n.d.	n.d.	1.85± 0.12	n.d.	n.d.	n.d.	n.d.	76.18± 1.72	10.14± 0.49	n.d.	n.d.	n.d.
Cichorium intybus L.	n.d.	n.d.	n.d.	n.d.	n.d.	n.d.	n.d.	n.d.	n.d.	n.d.	n.d.	100± 0	n.d.	n.d.	n.d.	n.d.	n.d.	0± 38.14	n.d.
Cirsium arvense (L.) Scop	n.d.	n.d.	n.d.	n.d.	n.d.	n.d.	n.d.	tr	n.d.	tr	n.d.	n.d.	n.d.	n.d.	n.d.	n.d.	n.d.	1.36± 0	n.d.
Consolida regalis S.F.Gray	n.d.	n.d.	n.d.	n.d.	n.d.	n.d.	n.d.	n.d.	n.d.	tr	tr	n.d.	n.d.	n.d.	n.d.	n.d.	n.d.	n.d.	n.d.
Crepis biennis L.	0.42± 0	1.41± 0.22	2.36± 0.07	n.d.	7.54± 0.51	1.46± 0.16	n.d.	2.5± 0.11	12.67± 0.18	3.17± 0.27	44.1± 0.79	n.d.	n.d.	19.05± 0.73	n.d.	n.d.	n.d.	n.d.	n.d.
Descurainia sophia (L.) Prantl	n.d.	n.d.	n.d.	n.d.	36.6± 0.54	n.d.	tr	n.d.	23.48± 1	14.67± 0.65	11.9± 0.79	n.d.	n.d.	n.d.	n.d.	n.d.	n.d.	n.d.	n.d.
Fumaria officinalis L.	n.d.	n.d.	n.d.	n.d.	10n.d.	tr	n.d.	n.d.	n.d.	n.d.	n.d.	n.d.	n.d.	n.d.	n.d.	n.d.	n.d.	n.d.	n.d.
Galinsoga parviflora Cav.	n.d.	n.d.	n.d.	n.d.	tr	n.d.	n.d.	n.d.	n.d.	46.38± 1.63	39.99± 0.45	n.d.	n.d.	n.d.	n.d.	9.43± 0.9	n.d.	n.d.	n.d.
Galium aparine L.	n.d.	n.d.	n.d.	n.d.	10n.d.	n.d.	tr	n.d.	n.d.	n.d.	n.d.	n.d.	n.d.	n.d.	n.d.	n.d.	n.d.	n.d.	n.d.
Lapsana communis L.	n.d.	n.d.	n.d.	n.d.	40.4± 1.49	n.d.	n.d.	n.d.	n.d.	21.42± 0.87	22.42± 0.45	n.d.	n.d.	n.d.	n.d.	3.95± 0.15	10.71± 0.58	n.d.	64.6± 1.7
Leonurus cardiaca L.	n.d.	n.d.	n.d.	n.d.	14.21± 0.9	n.d.	n.d.	2.56± 0.27	1.35± 0.19	6.84± 0.86	9.47± 0.47	n.d.	n.d.	n.d.	n.d.	n.d.	n.d.	n.d.	n.d.
Lepidium ruderale L.	n.d.	n.d.	n.d.	n.d.	17.04± 0.21	n.d.	n.d.	3.47± 0.17	n.d.	26.31± 0.74	50.74± 1.31	n.d.	n.d.	n.d.	n.d.	n.d.	n.d.	n.d.	n.d.
Melilotus albus Med.	n.d.	n.d.	n.d.	n.d.	49.68± 0.6	n.d.	n.d.	n.d.	n.d.	50.24± 0.61	n.d.	n.d.	n.d.	n.d.	n.d.	n.d.	n.d.	n.d.	n.d.
Potentilla argentea L.	n.d.	n.d.	n.d.	n.d.	n.d.	tr	n.d.	n.d.	n.d.	36.63± 0.89	28.86± 0.51	n.d.	n.d.	n.d.	n.d.	n.d.	n.d.	34.14± 0.39	n.d.
Silene latifolia alba (Mill.) Greut. et Burdet	n.d.	n.d.	n.d.	n.d.	n.d.	tr	n.d.	tr	n.d.	n.d.	tr	n.d.	n.d.	n.d.	n.d.	n.d.	n.d.	n.d.	n.d.
Sisymbrium loeselii L.	n.d.	n.d.	n.d.	n.d.	n.d.	2.73± 0.22	n.d.	n.d.	10.79± 0.41	62.85± 0.2	n.d.	n.d.	n.d.	n.d.	n.d.	4.37± 0.07	n.d.	n.d.	n.d.
Stellaria media (L.) Vill.	n.d.	n.d.	n.d.	n.d.	n.d.	tr	n.d.	tr	n.d.	n.d.	tr	n.d.	n.d.	n.d.	n.d.	n.d.	n.d.	n.d.	n.d.
Taraxacum officinale agg.	n.d.	n.d.	n.d.	n.d.	n.d.	n.d.	n.d.	n.d.	n.d.	15.16± 0.75	n.d.	n.d.	n.d.	n.d.	n.d.	64.56± 1.03	n.d.	17.04± 0.69	n.d.
Thlapsi arvense L.	n.d.	n.d.	n.d.	n.d.	n.d.	n.d.	n.d.	tr	n.d.	10n.d.	n.d.	n.d.	n.d.	n.d.	n.d.	n.d.	n.d.	n.d.	n.d.
Tripleurospermum inodorum (L.) Schultz-Bip.	n.d.	n.d.	n.d.	n.d.	n.d.	7.37± 0.29	n.d.	14.17± 0.36	2.7± 0.37	58.71± 1.27	13.39± 1.17	n.d.	n.d.	n.d.	n.d.	n.d.	n.d.	n.d.	n.d.
Urtica dioica L.	n.d.	n.d.	n.d.	n.d.	17.96± 0.35	n.d.	n.d.	n.d.	n.d.	18.21± 0.25	17.21± 0.28	n.d.	n.d.	n.d.	n.d.	34.96± 1.12	4.15± 0.24	6.2± 0.44	n.d.
Viola arvensis Murray	n.d.	n.d.	n.d.	36.44± 0.95	57.29± 0.5	n.d.	n.d.	tr	n.d.	n.d.	n.d.	n.d.	n.d.	n.d.	n.d.	n.d.	n.d.	n.d.	n.d.
